# Olaparib in an ovarian cancer patient with end-stage renal disease and hemodialysis

**DOI:** 10.1007/s00280-023-04514-x

**Published:** 2023-03-22

**Authors:** Joanna Baum, Daniel Zickler, Juliane Bolbrinker, Rolf Richter, Elena Ioana Braicu, Jacek Grabowski, Jalid Sehouli

**Affiliations:** 1 European Competence Center for Ovarian Cancer (EKZE), Department of Gynecology with Center for Oncological Surgery, Charité—Universitätsmedizin Berlin, corporate member of Freie Universität Berlin and Humboldt-Universität zu Berlin, Campus Virchow Klinikum, Augustenburger Platz 1, 13353 Berlin, Germany; 2 Department of Nephrology and Internal Intensive Medicine, Charité—Charité—Universitätsmedizin Berlin, corporate member of Freie Universität Berlin and Humboldt-Universität zu Berlin, Campus Virchow Klinikum, Augustenburger Platz 1, 13353 Berlin, Germany; 3 Institute of Clinical Pharmacology and Toxicology, Charité—Universitätsmedizin Berlin, corporate member of Freie Universität Berlin and Humboldt-Universität zu Berlin, Charitéplatz 1, 10117 Berlin, Germany

**Keywords:** Olaparib, PARP inhibitor, Maintenance therapy, Ovarian cancer, End-stage renal disease, Hemodialysis

## Abstract

**Purpose:**

For patients with severe renal impairment (CrCl ≤ 30 ml/min) or end-stage renal disease (ESRD), olaparib intake is not recommended as the pharmacokinetics and safety of olaparib have not been evaluated in this patient group. Therefore, this valuable patient group is generally excluded from poly(ADP-ribose) polymerase inhibitor (PARPi) therapy. Here we report the pharmacokinetics (PK), efficacy, safety and tolerability of olaparib capsules 200 mg BID in a patient with recurrent epithelial ovarian cancer (EOC) and ESRD requiring hemodialysis.

**Methods:**

Blood and dialysate samples of the patient were collected on a dialysis and non-dialysis day. Olaparib total plasma concentrations were determined through high-performance liquid chromatography with tandem mass spectrometric detection. Actual scheduled sample times were used in the PK analysis to determine multiple dose PK parameters at steady state.

**Results:**

Maximum concentration was achieved 1.5 h after drug administration on non- dialysis and after 1 h on dialysis day. The steady-state trough concentration and the maximal plasma concentration were similar on dialysis and non- dialysis day. On non-dialysis day, the AUC_ss_ was 30% higher (24.0 µg.h/mL vs. 16.9 µg.h/ml) than on dialysis day. The plasma clearance CL_ss_/F was lower on non-dialysis day. Olaparib was not detectable in the dialysate samples.

**Conclusion:**

A total dose of olaparib 200 mg BID capsule formulation was well tolerated by our patient with ESRD and hemodialysis. Moreover, this maintenance therapy led to 16 months of progression free survival. Further trials on PARPi therapy in patients with hemodialysis are warranted.

## Introduction

In the last years, the concept of maintenance therapy has been introduced and constantly developed in advanced primary and relapsed high- grade epithelial ovarian cancer (EOC). Olaparib is the first-in-class poly(adenosine diphosphate-ribose) polymerase-inhibitor (PARPi) and was proved highly beneficial for platinum-sensitive EOC patients in phase III trials [1, 2]. More recently, its approved application range was extended [3]. It can be applied as maintenance therapy for primary advanced, platinum- sensitive EOC with evidence of a Breast Cancer gene (BRCA) mutation [2–4]. In case of homologous recombination deficiency (HRD) in the tumor, a combined therapy with bevacizumab can be initiated [3]. Furthermore, olaparib may also be applied in the relapse situation without any proof of mutation [3].

Currently, for patients with renal impairment, the application of olaparib is based on creatinine clearance (CrCl): for patients with mild renal impairment (CrCl 51–80 ml/min) no dose adjustments are required, for moderate renal impairment (CrCl 31–50 ml/min) a dose reduction to 200 mg BID for tablet formulation and 300 mg BID for capsule formulation is recommended [3]. For patients with severe renal impairment (CrCl ≤ 30 ml/min), or end-stage renal disease (ESRD), olaparib intake is not recommended as the pharmacokinetics and safety of olaparib have not been evaluated in this patient group.

Here we report the pharmacokinetics (PK), efficacy, safety and tolerability of olaparib capsules 200 mg BID in a patient with recurrent EOC and ESRD requiring hemodialysis.

## Methods

### Patient’s characteristics

A 77-year-old patient was diagnosed with a FIGO IIIC stage high-grade serous ovarian cancer in October 2013. Preoperatively, the patient did not have any preexisting comorbidities and did not take any medication. She underwent a primary complete cytoreduction on 28th October 2013 consisting of en- bloc resection of uterus and adnexa, resection of the rectum with an end- to- end anastomosis, peritonectomy of the pelvis and paracolic gutters, partial resection of the right diaphragm, omentectomy, appendectomy, para- aortic and pelvic lymph node sampling, ureterolysis as well as a subtotal peritonectomy. A tumor of less than 0.5 cm remained in the hilum of the liver. Due to diffuse bleeding, the patient received 1.5 l of colloidal solution, 19 units of fresh frozen plasma and four units of red cell transfusion during the operation. Intraoperatively, a renal ESRD of unknown cause was diagnosed, reducing the glomerular filtration rate from preoperatively > 90 l/min to postoperatively < 15 l/min.

Since that time, the patient required hemodialysis three times a week. Dialysis was performed via an upper arm cephalic vein arteriovenous fistula with a blood flow of 250 ml/min and a dialysate flow of 500 ml/min. A High-Flux dialysis filter (Revaclear 400^©^) and a Gambro dialysis machine (AK 200^©^) were used. Unfractionated heparin was administered at a rate of 1000 units/hour after a starting bolus of 1000 IE. The total ultrafiltration rate amounted to 2000 ml with a dialysis session lasting for four hours. Subsequently, an adjuvant chemotherapy with carboplatin 100 mg absolute dose and paclitaxel 175 mg/m^2^ was initiated in January 2014. However, chemotherapy was discontinued after two cycles as the patient developed delirium with fluctuating cognitive deficiencies, most likely in the context of paraneoplastic syndrome, with spontaneous recovery during the course of time. In August 2015, an embolic stroke of unknown source was diagnosed in the middle cerebral artery and the posterior inferior cerebellar artery leaving the patient with a light ataxia of gait. At the same time, the first platinum-sensitive recurrence was diagnosed with a peritoneal and lymph nodal tumor spread. Due to hemodialysis, she received five cycles of carboplatin monotherapy with a reduced absolute dose of 300 mg per cycle. A partial remission was achieved. In April 2016, maintenance therapy with olaparib capsules with a reduced dose of 200 mg BID was started and taken regularly by the patient for 16 months until progression of disease was diagnosed.

### Analysis

In October 2016, after informed consent of the patient was obtained, venous blood and dialysate samples were drawn from the patient to determine the plasma concentration of olaparib on one dialysis and one non-dialysis day. On the dialysis day, blood samples were collected prior to, 1, 1.5, 2, 3, 4, 5.5, 6 and 8 h after olaparib intake. Hemodialysis started 1.5 h after olaparib intake and lasted for four hours. In doing so, dialysate samples were taken at 1.5, 2.5, 3.5, 4.5 and 5.5 h after olaparib intake. On the non-dialysis day samples were taken prior to, 1, 1.5, 2, 3, 4, 6, and 8 h after olaparib intake. Blood samples were centrifuged for 10 min and stored at − 20 °C.

Plasma concentrations of olaparib were determined using a validated reversed-phase, high-performance liquid chromatography method with TurboIonSpray^®^ tandem mass spectrometric detection in positive ion mode (HPLC–MS/MS). Following the addition of deuterated internal standard ([^2^H_8_] olaparib), plasma samples (100 µL) were diluted with water and subjected to manual solid phase extraction on Phenomenex StrataTM-X cartridges. Following elution with acetonitrile and evaporation to dryness, the extracts were reconstituted with HPLC mobile phase (500 µL) and chromatographed on a Waters Xterra^®^ Phenyl 3.5 µm analytical column (50 × 2.1 mm I.D.) eluted at 0.2 mL/min with 1 mM pH3 ammonium formate buffer/acetonitrile (73/27 v/v). Olaparib and internal standard were detected by TurboIonSpray (positive mode) mass spectrometric detection, monitoring ions 435.2 → 281.1 and 443.0 → 281.1 respectively. Calibration curves for Olaparib, prepared in human plasma, were analyzed alongside each batch of samples and the data analyzed using linear regression employing 1/x weighting. The limit of quantification of the assay was 0.5 ng/mL with linearity established over two calibration ranges (0.5–500 ng/mL and 0.02–20 µg/mL). The performance of the assay was monitored throughout use by the inclusion of quality control samples in all bioanalytical runs performed. Assay precision and bias were shown to be within acceptable limits (< 20% at the lower limit of quantification and < 15% at all other concentrations) during study sample analysis.

Dialysate samples were assayed alongside plasma samples using the low range plasma method described previously with the addition of quality control samples prepared in phosphate buffered saline as a non-proteinous surrogate matrix for dialysate. Both the precision and bias of these quality control samples were again shown to be within acceptable limits during study dialysate sample analysis.

Plasma concentration–time data was analyzed using Phoenix^™^-WinNonlin^®^ v6.3 via non-compartmental analysis. Actual scheduled sample times were used in the pharmacokinetics (PK) analysis to determine multiple dose PK parameters at steady state, namely time to reach maximum plasma concentration (t_max,ss_), maximal plasma concentration (C_max,ss_), steady-state trough concentration (C_min,ss_), area under the concentration- time curve (AUC) over 12 h of drug administration (AUC_ss_), AUC until the last measurable concentration after eight hours (AUC_last,ss_) and apparent plasma clearance following oral administration of the drug (CL_ss_/F). Since samples for bioanalysis were collected up to only 8 h after dosage, concentrations at 12 h after drug administration were estimated based on the terminal elimination rate constants. They were determined based on at least 4 data points.

## Results

### Safety and tolerability

Olaparib intake began in April 2016 and treatment continued for 16 months without interruption. Treatment was stopped after 16 months due to progressive disease. Mild nausea and fatigue were present at the beginning of therapy and subsided after a few weeks. No other toxicities were reported.

### Pharmacokinetics

**Fig. 1:**
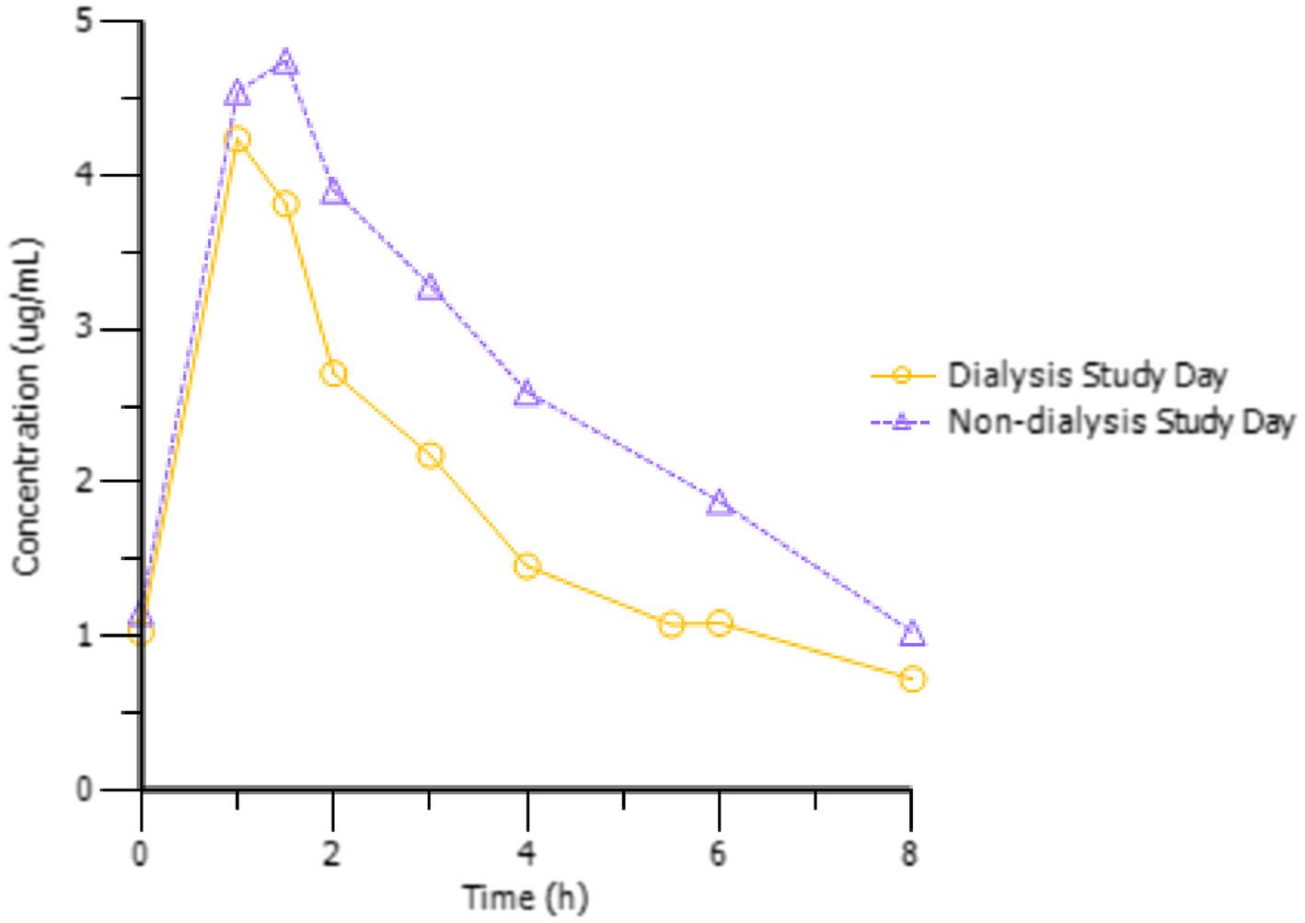
Multiple dose plasma concentration profiles at steady-state of olaparib in a patient with ESRD on one dialysis and one non-dialysis day

The plasma concentration time profiles of olaparib for the patient with ESRD on dialysis and non-dialysis day are shown in Fig. 1. Although the individual plasma concentrations were lower on dialysis-day compared to non-dialysis day, the olaparib doses 1 h after intake, steady-state trough concentrations and maximum plasma concentrations were similar.

Multiple dose PK parameters of the patient at steady-state of olaparib at 200 mg capsule BID on dialysis and non-dialysis day are summarized in Table 1. Normally, the patient was dosed with olaparib at 11 a.m. and 10 p.m. During the days of sampling, the patient took olaparib at 7 a.m. Hence, the observed pre-dose concentrations reflect steady state concentrations at 9 h post dose and would therefore slightly overestimate the steady state trough concentrations. The C_min,ss_ values in Table 1, which were estimated based on the elimination rate constants post distribution, are more representative of steady-state trough concentrations.

**Table 1:** PK parameters on dialysis and non-dialysis day

Day	t_max,ss_ (h)	C_max,ss_ (µg/mL)	AUC_ss_ (µg.h/mL)	AUC_last,ss_^a^ (µg.h/mL)	Pre-dose concentration (µg/mL)	C_min,ss_^b^ (µg/mL)	CL_ss_/F (L/h)
Dialysis	1	4.23	16.9	14.8	1.03	0.726	11.8
Non-dialysis	1.5	4.74	24.0	21.2	1.16	1.03	8.33

^a^AUC_last,ss_ = AUC_(0–8)_

^b^Estimated

Maximum concentration was achieved 1.5 h after drug administration on non- dialysis and after 1 h on dialysis day. The steady-state trough concentration and the maximal plasma concentration were similar on dialysis and non- dialysis day. On non-dialysis day, the AUC_ss_ was 30% higher (24.0 µg.h/mL vs. 16.9 µg.h/ml) than on dialysis day. The plasma clearance CL_ss_/F was lower on non-dialysis day. Olaparib was not detectable in the dialysate samples.

## Discussion

Data regarding maintenance therapy with olaparib in patients with ESRD is very limited. To the authors knowledge, this is the first report on the PK of olaparib in a patient with ESRD on hemodialysis.

On dialysis day, olaparib levels were slightly below the levels of the non-dialysis day. On non-dialysis day, C_max,ss_ was approximately 10% higher and the AUC_ss_ was 30% higher than on dialysis day, possibly due to enhanced elimination during the dialysis session. However, it must be emphasized that even on non-dialysis day the geometric mean C_max_ and AUC_ss_ value of our patient was similar to the values observed in patients with normal renal function receiving the same dose [5]. In detail, in the phase I analysis of Fong et al. 17 patients received 200 µg of olaparib where a C_max,ss_ of 5.62 µg/ml and a median AUC_0–12 h_ of 33.3 µg.h/ml was measured [5]. In contrast to that, in our patient C_max,ss_ was 4.74 µg/ml and the AUC_ss_ 24.0 µg.h/mL. This comparison shows that C_max,ss_ and AUC_ss_ of olaparib in our patient did not exceed the mean values determined in the phase I study and hence supports the safety aspects of our application. However, when applying the optimal dosage of 400 µg olaparib BID to patients with normal renal function, the C_max,ss_ was 7.65 µg/ml and the AUC_0–12 h_ 44.9 µg.h/ml [5]. As our patient did not reach those optimal concentration levels of olaparib it can be concluded that our patient might even have been underdosed.

If the olaparib plasma concentrations observed in this patient are typical of patients with ESRD on hemodialysis in general, then ESRD on hemodialysis appears to have an effect on the PK of olaparib. It is known that accumulated uremic toxins associated with chronic kidney disease without hemodialysis can downregulate cytochrome P450 (CYP) 3A expression and activity in the gut and in the liver [6]. Available trials show that olaparib is cleared with a proportion of 84% through hepatic metabolism based on oxidation via CYP 3A4/5 [7]. Hence, a further explanation for the higher exposure to olaparib observed in patients with renal impairment compared to patients with ESRD and hemodialysis is that it might be caused by uremia and the associated downregulation of these enzymes [7]. In line with this, a study of 38 patients with normal renal function, mild and moderate renal insufficiency and intake of a single oral 300 mg dose of olaparib tablet formulation showed an increase of AUC and C_max_ by 24% and 15% respectively for mild and 44% and 26% for moderate renal insufficiency compared to patients with normal renal function [8]. In contrast, our patient with ESRD and hemodialysis presented with AUC and C_max_ similar to patients with normal renal functions.

We did not find a significant quantity of olaparib in the dialysate fluid. As we noted lower olaparib AUCs on dialysis day, it seems likely that olaparib was actually filtered, but could not be detected, due to extreme dilatation of the metabolite and insufficient assay sensitivity. One has to keep in mind, that during a 4 h dialysis session, 120 L dialysate are produced, thus olaparib concentrations in this amount of dialysate are extremely difficult to detect. Another possible explanation is that olaparib was attached to the dialysis filter and was thus removed from the blood. Either way, it seems likely the dialysis session contributed to olaparib elimination.

One limitation of the study is the that only one patient with ESRD and hemodialysis was treated with olaparib. Nevertheless, this treatment led to a PFS of 16 months. In comparison, the median PFS of BRCA- mutated patients with normal kidney function receiving a standard dose of 400 mg BID is 11.2 months [9]. Moreover, the treatment was well tolerated by the patient. Generally, common mild adverse reactions of CTCAE grade 1 and 2 include fatigue and gastrointestinal symptoms such as nausea, vomiting, abdominal pain and diarrhea [1]. The prevalent adverse incidence of grade 3–5 is anemia [1]. Our patient had only mild nausea and fatigue at the beginning of the treatment.

Another limitation is that the patient received olaparib capsules which were gradually replaced by the more comfortable tablet formulation. Although both formulations differ in dosage and bioavailability, the substance and therefore the mechanism of action remain the same so that parallels can be drawn.

The peculiarity of this study is the focus on patients with ESRD and hemodialysis in an ovarian cancer setting. Those vulnerable patients are often excluded from medical studies. Our patient suffered from perioperative acute kidney injury (AKI) with the need of hemodialysis. Perioperative AKI constitutes a common risk in gynecological surgeries with an overall prevalence of 13% [10]. Thereof, 15% develop a Risk, Injury, Failure, Loss and End- stage renal failure (RIFLE) with 3% of those patients requiring hemodialysis [11]. Those numbers show the high relevance of this topic and the need to focus on this special patient group.

In conclusion, a total dose of olaparib 200 mg BID capsule formulation was well tolerated by our patient with ESRD and hemodialysis. Moreover, this maintenance therapy led to a 16 months PFS in our patient. Still, an optimal dose of olaparib for patients with ESRD on hemodialysis cannot be defined at this point. The optimal dose should be rather specified individually depending on adverse reactions. In this context, vomiting, nausea and fatigue are the most common causes for dose reductions or interruptions. Given the increasing number of patients receiving PARPi, further trials in patients with hemodialysis are warranted.

## Data Availability

The dataset generated during and/or analyzed during the current study are available from the corresponding author on reasonable request.
